# Adjustment to short-term imprisonment under low prison staffing

**DOI:** 10.1192/bjb.2020.2

**Published:** 2020-08

**Authors:** Sarah O'Connor, Zoe Bezeczky, Yvonne Moriarty, Natasha Kalebic, Pamela J. Taylor

**Affiliations:** 1 Division of Psychological Medicine and Clinical Neurosciences, School of Medicine, Cardiff University, UK; 2 Centre for Trials Research, College of Biomedical & Life Sciences, Cardiff University, UK

**Keywords:** Short-term prisoners, prison experience, adjustment to imprisonment, prison routine, prison milieu

## Abstract

**Aims and method:**

To understand experience of early imprisonment in one prison under low staffing levels. A researcher, independent of the prison, interviewed each prisoner soon after reception and 3–4 weeks later. The first question of the second interview was: ‘I’d like to start by asking you about your experience of the last 3–4 weeks in prison'. Data are verbatim answers to this. Narratives were brief, so responses from all 130 participants were analysed, using grounded theory methods.

**Results:**

The core experience was of ‘routine’ – characterised by repetitive acts of daily living and basic work, and little reference to life outside prison – generally resolved passively, towards boredom and ‘entrapment’.

**Clinical implications:**

This ‘routine’ seems akin to the ‘institutionalism’ described in the end days of the 1960s’ mental hospitals. In an earlier study of similar men at a similar stage of imprisonment, under higher staff:prisoner ratios, experience was initially more distressing, but resolved actively and positively, suggesting that staff loss may have affected rehabilitative climate.

More men are imprisoned in England and Wales than in any other Western European country.^1^ Many prisoners have at least one mental disorder,^2^ but few are transferred to a healthcare setting for treatment, so most treatment and most programmes for offending behaviour are delivered in prison. Any impact of these is likely to be affected by the prison milieu. Studies have shown that people seem to be particularly vulnerable during early imprisonment, especially to self-harm,^3,4^ although there is widespread evidence of adjustment, including improvement of mental state over 4–6 weeks.^5,6^ Since these studies, however, there have been substantial cuts to prison staffing in England and Wales – about 40% in publicly run prisons since 2013.^7^ Over the same period, prisoner suicide, self-harm and violence rates have risen.^8^ Austerity in public service delivery is far from unique to the UK, so it is important to understand day-to-day prisoner experience and adjustment under reduced staffing. In one prison in public management, for example, prison officer numbers had been cut from 200 in 2013 to 148 in 2016, while the number and type of prisoners had remained more or less constant; there were 763 prisoners, of whom 52% were unsentenced or serving sentences of less than 6 months, in 2013, compared with 770, of whom 57% were similarly short-term, in 2016.^9,10^ Our research question was: How did new receptions in this prison affected by staff cuts experience day-to-day living during the first 4–5 weeks of a new imprisonment?

## Method

### Overview

Applying a grounded theory approach, a researcher employed independently of the prison recorded prisoners’ responses to an open question about experience of the current imprisonment. This study was designed to stand alone, although it was embedded in a larger study, a randomised controlled trial of a 9-group intervention for alcohol misusing prisoners.

### Ethics

The overarching study, including the qualitative components, had ethical approval from the National Health Service Health Research Authority National Research Ethics Service (NRES) Committee East of England – Essex (IRAS ref. 140458; REC ref. 14/EE/0046). Information leaflets about the research were made available to all prisoners on reception. Each potentially eligible prisoner was approached by the researcher, who explained the research to each man in private, assured him that neither his treatment in the prison nor in the criminal justice system more widely would be affected by a decision to participate or not and answered any questions about the research. Confidentiality was assured, except with reference to any specific information about intent or plan to harm self or others or to escape from the prison. Consenting men were asked to sign a consent form. Each man had at least 24 h to reflect before full participation and could withdraw at any time if he changed his mind about the research. The rate of refusal to participate was below rates reported in similar studies (P. J. Taylor, personal communcation, 2019), but still about one in five men approached for screening refused and 27 (11%) of the eligible men initially agreeing to participate subsequently withdrew.

### Sample

Participants were recruited between June 2014 and August 2016, from men newly received into one UK prison. In a two-stage screening process, the first stage was to identify, from records, all men likely to be in prison on this occasion for under 12 months (short-term prisoners) but to remain in this prison for at least 1 month, thus likely to acquire sufficient experience of the environment to be able to describe living in the prison and adjusting to it rather than talk about receiving a prison sentence *per se*. About 1 week after reception, a researcher invited these men to meet her to explore willingness to participate, to take formal consent from those willing and to complete a second, face-to-face screen for alcohol^11^ or drug^12^ misuse. All 238 consenting men screening positive for substance misuse were included; 197 remained for interview on a subsequent day up to a week later about pre-prison life experiences and their mental state. Between 3 and 4 weeks later, those still in prison and consenting were interviewed about their experience of this imprisonment and their mental state was re-evaluated. Data from the opening of this second interview are analysed in the study we report here.

For the trial, the men were randomised to receive either standard prison regime alone or prison regime and a 3-week group programme between these two interviews. The group programme included motivational work and self-management skills development, delivered by clinical psychologists from a local health board. Both intervention and treatment-as-usual men were included in this qualitative work.

### Procedures

All interviews were conducted in private, by the same researcher on both occasions for each man. The data for this study were responses to the opening question of the second interview: ‘I'd like to start by asking you about your experience of the last 3–4 weeks in prison’. After this, only simple, neutral prompts were used to encourage the men to talk freely about this, for example ‘go on’, ‘tell me more’. Each prisoner's responses were documented contemporaneously and any abbreviated words or phrases written up in full immediately after the interview was complete. The interviews were not audio-recorded; audio-recording is commonly discouraged in grounded theory work and external researchers are not generally permitted to take recording equipment into prisons. Once each man had said everything that came to his mind, unprompted by us, about this imprisonment, he was asked some specific, supplementary questions about aspects of the imprisonment, including how much time he spent out of his cell, whether he had work, education and/or outside visitors, and whether he got on with prison staff and other prisoners. In turn, we were able to access independent reports on this prison from Her Majesty's Inspectorate of Prisons.^9,10^ These two separate sources of data allowed some *post hoc* consideration of the extent to which reported experiences fitted with actual activities on the one hand and general prison conditions on the other.

### Data analysis

Anonymised, free narrative data were analysed in two batches – control- and intervention-arm men – by researchers blind to trial-arm membership and without reference to answers to specific questions about this imprisonment. This was to allow for the possibility that participating in groups as part of the trial affected the standard prison experience. The narratives tended to be short, a third of them not more than three sentences, so we decided to analyse all of them rather than defining the sample size by data saturation as would be more usual in a study of this kind. We used a grounded theory approach to analysis.^13,14^ The first narrative was examined, and categories of information contained in it extracted into a table, as far as possible labelling each category with a word or phrase used by the participant, with the supporting evidence of the full quotation. The second narrative was analysed in a similar way, using already identified categories where possible and adding new ones as appropriate. Two of us analysed the first 10 narratives masked to each other, then compared the ratings. Differences between us lay only in the extent to which we had listed each item as a separate category of routine – for example ‘having food’ as a common term for taking meals rather than listing each meal as a separate category. It was agreed that even the smallest of categories would be listed initially, after which both extractions were in full agreement.

We then completed first-level category identification from each batch separately (see Supplementary Tables 1 and 2 available at https://doi.org/10.1192/bjb.2020.2). It was apparent that very similar categories of experience were emerging, regardless of trial arm, so data from all the men were combined for further analysis. Using constant comparative analysis, higher-order categories were allowed to emerge, and then a core category, which best encompassed all the other categories.

## Results

### Sample characteristics

In total, 130 men provided valid interviews. Given the sample size, we have not tabled each man's personal characteristics for context but provide the following summary. Their mean age was 30 years (s.d. = 7.9). Most (101/130) had been in prison before, with a mean total time spent in prison, after adding their various remands and sentences together, of just over 5 years (5.17, s.d. = 5.65). Two-thirds had mental health concerns (87/130), over a quarter physical health concerns (36/130) and screening confirmed that all were struggling with problem substance use.

### The core experience of this imprisonment

The core category or concern was of ‘routine’ within the prison. The most repeated elements were activities of daily living (‘got up’, ‘made a cuppa’, ‘food’, ‘fag’, ‘nap’, ‘TV’, ‘association’ (when prisoners are allowed to mingle freely out of their cells), ‘cleaning’), with most men making some reference to at least one of these. Most of these activities were just listed to us – without further comment – but in a few cases comments were explicitly negative – ‘do a bit of work, well I say work, fuck around on the computer. I'm wasting time […] really’ (141); ‘I'm fed up of TV’ (230) (the number shown in parentheses indicates the particular man making the statement). A few men mentioned going to the gym or taking other forms of exercise, and a few were explicit about not doing so. Other activities sought by the men as part of a healthy routine but which required more initiative met mostly with limits and frustration. These activities were work, education and courses. Few men reported attending education or courses, but most were preoccupied with seeking work – ‘my brain needs to focus on something’ (217). More than half reported actually working, although often repetitive cleaning or prison maintenance, with some explicitly objecting to this: ‘I don't want to just work for the prison’ (154). Others were explicit about the frustrations of trying to get ‘real work’: ‘I'm frustrated because I didn't get a job’ (217). Many seemed accepting, coming back to the concept of routine: ‘You get into a routine and tell yourself it's not forever’ (215).

This dreary routine also seemed to encompass the men's experience of the outside world. Few men volunteered reference to family or friends, and most of these only in terms of ‘routine visits or phone calls’. The few men who referred to outside events with emotion were all negative: ‘they wouldn't let me go to my Dad's funeral; I was a bit upset’ (120); ‘Nan passed away […] someone came from the chapel […] he asked if I was alright and if I was going to do anything stupid while I was in here’ (211).

### The model of adjustment to this imprisonment

The men all felt some sense of movement over the 3 weeks in relation to this ‘routine’. Two directions of resolution were apparent. The stronger was passive movement towards feeling ever more trapped or ‘banged up’. The weaker, experienced at all by very few, was of ‘being busy’ and even of ‘time flying’.

Passive resolution was characterised by comments such as: ‘The same stuff, day in day out, it just does your head in’ (100); ‘spend all my time sweltering in my cell’ (219); ‘banged up most of the time’ (109). For a few, though, even this restrictive routine provided a kind of stability: ‘I'm settled now. Been in 10 times and got my routine now’ (128); ‘I like the routine of prison’ (253).

The very few men who described more active movement towards ‘being busy’ and ‘time flying’ were not only looking for ‘new opportunities’, but considered that they had found them: ‘it's busy, and I like to keep busy’ (106); ‘time goes quicker now I'm doing stuff’ (117). Just two men stood out as different because they specified that they themselves were trying to help others, which gave them a sense of purpose: ‘I'm also the smokers’ champion – I give people advice on coping strategies, just like being a listener really’ (134); ‘I've been cleared to be a prisoner listener. History of self-harm, so surprised, didn't ever think I would. Look forward to starting that’ (153). Further, when these more positive things happened, prison staff were invariably also seen in a positive light and as helping them to move in a positive direction.

### Barriers to and facilitators of adjustment

In this model of adjusting to imprisonment, the men volunteered particular barriers and facilitators as affecting direction of movement towards being trapped and bored or towards being busy. These broadly fell into two types – personal or prison issues.

#### Personal issues

The few personal issues raised relating to life outside prison were almost invariably described as problems, leaving the men feeling more restricted and trapped: ‘I'm stressing a lot, thinking I'm a parent, shouldn't be here, I should be out there looking after my missus and kids’ (102).

Reports of the impact of relationships in prison were more mixed. Some liked their relationships with other prisoners and thought they helped pass the time positively: ‘chill out with the boys and have a chat, the boys are all good in here’ (103). Most were more negative, with ‘routine irritations’ beyond their control promoting a negative path towards an increasing sense of entrapment: ‘me and my cell mate just end up bugging the shit out of each other’ (100); ‘It's hell in here – kicking doors, bunch of kids’ (207). There was an occasional report of loss of an in-prison attachment as a stressful ‘outside-prison’ issue: ‘I was in with my other mate, but he went to [another] prison. I'm gutted. I won't be seeing him for three years – that's how long he's got left. I'll have to do another sentence to see him’ (141).

Another major personal issue frequently referred to was ill health. Most comments indicated that this was a real barrier to progress and left individuals feeling restricted. Occasionally, these were in the form of a simple statement of fact: ‘my liver is fucked’ (112); ‘I got a diagnosis. PTSD’ (230). Sometimes state of health was a more explicit barrier: ‘Won't let me go to the gym because of my blood pressure’ (101); ‘Sleeping mostly. My head is shot’ (223). Six men, though, thought prison was helping or could help their health specifically: ‘No, it's brilliant. I feel better and put a bit of weight on’ (138); ‘I've seen mental health today – let them know my frustrations. She is going to help me’ (134).

#### Prison system issues

The prison system issues that most felt frustrated by were the ‘routine blocks’, or barriers, to their efforts which left them trapped in their poor health, boredom and numbing routine. Very occasionally, this was attributed to staff personally – ‘Staff don't care’ (238) – but mostly to the system. This was of particular concern in relation to health: ‘I'm waiting to see the dentist. Remember I had toothache last time you came [3 weeks before]? Well I've got an abscess now. I asked to see the dentist, but I've not heard back’ (147); ‘I still haven't seen mental health’ (222); ‘I was pissing blood and passed kidney stones on Monday. There is no help in here’ (148). Prison issues posing barriers to occupation were commonly described, with most wanting to be productive but being frustrated in their efforts: ‘I've applied for everything, I'll do anything’ (262); ‘You read the prison policies and they say you must work and I'm here begging for it. I've spoken to the officers […] I've put three apps [applications] in so far. I said I would kick off in a week if I didn't get something but my partner said it's not worth it’ (217); ‘You don't seem to get anywhere when you put the applications in – we made a complaint but I haven't heard anything about that either’ (247); ‘I think the system is designed to break you’ (156). Prison-system problems were thus generally seen as frustrating recovery and a direct barrier to progress.

## Discussion

‘Routine’ is, by definition, made up of a series of repeated, expected actions. In some form, it is ubiquitous among human beings. It may be imposed in order to influence behaviours. Institutions, almost by definition, impose routines, whether deliberately or otherwise, so it may seem unsurprising that men put routine at the core of their experience of being in prison. The routine that most men reported, however, was impoverished and seemed comparable to reports from the end days of the big ‘asylums’ for people with mental disorder, in which the patients tended to become as impoverished as their environment.^15–17^ Wing^18^ subsequently emphasised that this could happen in the community too if resources were limited. A difference between the patients described by Wing and colleagues and these prisoners is that none of these prisoners had enduring psychotic illness, so it is possible that they were less vulnerable. A few prisoners welcomed the basic, limited repetitiveness of the experience and a very few found positive ways through the system. Most were explicit about finding the limitations frustrating and being unable to affect their situation. To what extent, however, could we rely on these accounts from, perhaps, disgruntled men and to what extent is this a consistent experience?

### Other evidence on the experience of being in this prison

There is an independent inspectorate of prisons for England and Wales (HM Inspectorate of Prisons), which conducts reviews of individual prisons as well as occasional thematic reviews of needs and services in them (https://www.justiceinspectorates.gov.uk/hmiprisons/). Fortuitously, an unannounced inspection of this prison took place in 2016, more or less at the same time as this research. The resultant report, despite referring to ‘a decent, hard-working staff group who had maintained good relationships with the men in their care, and had done well to keep the prison stable through some challenging times’ (p. 5), highlighted how low staffing levels had affected the responsiveness of staff to the needs of the men in the prison.^10^ In 2016, for example, only 16% of prisoners’ call bells were responded to within 5 min, compared with 39% in 2013; timetabled activities were run less often, application response rates fell from a 59% within 7 days in 2013 to 31% in 2016, and only 5% of men reported spending more than 10 h out of their cells in 2016 but 10% in 2013, all significant differences. This all fits with the limitations that the men in our sample were citing. It indicates that the prison milieu may be subject to substantial changes over time. This has implications for all prisoners and their chances of ‘reform’. From a trialist's perspective, it is clear that ‘treatment as usual’, the traditional standard against which psychosocial interventions are evaluated, must be measured in some detail in order to understand its meaning and potential impact. For clinical and criminal justice system practice, staff should be aware of the potential impact of the milieu on what they can deliver.

### How prisoner experience after staff cuts compares with experience at a better staffed time

We were able to consider the model of prisoner experience and adjustment for the years 2014–2016 in the context of data we collected in a similar way from similar men in this prison (and another in South Wales) in 2007–2008, before the prison staff cuts.^19^ In that study, narratives were much longer and richer, to the extent that we had clear data saturation (no new categories of information emerging) after just 20 cases. This in itself fits with the possibility that the later sample of men were, indeed, already so restricted by their ‘routine’ that they were less engaged in thinking and talking about themselves and their experiences. The core concern of these similar men in prison during the better staffed period was of the losses inflicted by the imprisonment and how awful the experience was. Although, even then, there was some passive resolution of this concern by ‘getting used to it’, most invoked a sense of active movement towards becoming ‘alright’, which meant feeling and getting better, making positive changes and developing good relationships. The men in the earlier sample spoke much more about how much they were missing people, freedom, information and other resources, whereas those in the current sample were much more focused on prison *per se*. The study samples were of different men, but as their age, sentences, prior experience of imprisonment and rates of reported mental health difficulties were so similar (the earlier sample is described in Taylor *et al*, 2010^5^), it is reasonable to consider that the difference in prison milieu and experience has had an impact.

Souza & Dhami,^20^ in a quantitative study of men in two English prisons at about the same time as our earlier study, also cited losses of family, friends and freedom as the hardest experiences reported by first-time and recurrent male prisoners, but also some resolution of problems through improving health and having opportunities for rehabilitation. They then argued that positive engagement or not was better explained by aspects of life before imprisonment and overall exposure to imprisonment than by prison security or regime. They could not envisage the extent of imminent cuts, and we must now question whether, for most prisoners, impoverished regimes force their focus onto prison conditions *per se* and limit capacity for concern about others and/or reflection and development.

### The advantages of richer routines

Behan^21^ examined the specific prisoner experience of educational programmes. Although some prisoners wanted to ‘better themselves’, gain new skills and prepare themselves for work on release, some used these programmes as a way of coping with their imprisonment, saying that it took their mind off their experience in prison and ‘killed time’. This use of education to better oneself or as a coping strategy resonates with the narratives given by our sample of men, some of whom were clearly wanting to develop their skills and abilities, whereas others just wanted to get out of the cell or the wing or simply fill the time. Behan suggests that attendance for experiences such as education may also give a greater sense of agency in being able to control their prison routine. Our men commonly found themselves frustrated and without agency because they wanted to be at education or, more likely, work and could not get there.

Nurse *et al*^22^ found, in a qualitative study of prison environment and mental health of prisoners and prison staff, that understaffing and a lack of activities led to increased stress and frustration among prisoners. The men in their sample, like those in ours, viewed any activity as important to ‘stimulate your mind’. Nurse and colleagues, however, found more tension between prisoners and staff than in either of our studies. Their data were, however, collected through focus groups rather than individually. It may be that prisoners feel more need to complain about staff when other prisoners are listening than when they can talk in private.

Reiter *et al*^23^ were wide ranging in their inquiries about prison experience, covering a broader range of prisoners and prison conditions than we did. All our prisoners were living in ‘ordinary locations’ within the prison during the study. Nevertheless, it is striking that in the relatively well-staffed Danish prisons of the Reiter study, men's experiences had more in common with those in the earlier of our studies. The Danish prisoners too seemed very aware of what they were missing by being in prison and, although making references to in-prison conditions, did not appear so mentally bound by their routine as the men in our ‘austerity prisoner sample’.

### Limitations

This was a qualitative component of a wider study and not set up as a primary open inquiry in its own right. Nevertheless, the question about experience of imprisonment was planned, open ended, consistent and posed before any other questions at the second interview after the men had had about a month of experience of imprisonment. The interviews were not audio-recorded, so the notes and final written record of the responses could not be checked except against each other, but as responses were generally not long or complex, we think it extremely unlikely that any key word or phrase was missed. The researchers collecting the data experienced some of the same frustrations in accessing the prisoners as the prisoners did in their daily living, which could have coloured data recording, but consistency on some key issues with the report published by HM Inspectorate of Prisons^10^ mitigates against this.

We have suggested that the dull, restricted, almost institutionalised experience of the men, so different from that of an earlier cohort, related to staff cuts. It is impossible to rule out other explanations completely, but the reduction in prisoner officer numbers from about 200 to fewer than 150 was the main observable change. Numbers and types of prisoner overall remained the same and there were only modest differences between research cohorts in likely key measures. Although all of the men in our later cohort screened positive for substance misuse so did 84% in the earlier cohort; 74% of the men in the earlier cohort had had prior experience of imprisonment, but so did 80% in the later cohort.

### Summary and implications

Focus on prison ‘routine’, which tended to leave prisoners feeling trapped, dominated short-term prisoners’ accounts of their time in this one UK prison at any time between July 2014 and August 2016. They did not raise concerns about the awfulness of the losses of family, friends and freedom incurred by imprisonment, as men in an earlier cohort had done, and hardly referred to the outside world. They rarely reported any positive resolution, which had been prominent among the men in the earlier study. The large change in staffing levels made a difference to the environment, and it seems that the core experience and adjustment of prisoners cannot be assumed to be a constant in such a context. Indicators that the later men were experiencing ‘institutionalism’, not apparent in an earlier, better staffed time, should concern those who fund and commission prisons.

## Supplementary material

For supplementary material accompanying this paper visit https://doi.org/10.1192/bjb.2020.2. click here to view supplementary material

## About the authors

**Sarah O'Connor** is a medical student in the Division of Psychological Medicine and Clinical Neurosciences, School of Medicine, Cardiff University, UK. **Zoe Bezeczky** is a research assistant in the Division of Psychological Medicine and Clinical Neurosciences, School of Medicine, Cardiff University, UK. **Yvonne Moriarty** is a research associate and the ABACus Trial Manager in the Centre for Trials Research, College of Biomedical & Life Sciences, Cardiff University, UK. **Natasha Kalebic** is a post-doctorate research assistant in the Division of Psychological Medicine and Clinical Neurosciences, School of Medicine, Cardiff University, UK. **Pamela J. Taylor** is Professor of Forensic Psychiatry in the Division of Psychological Medicine and Clinical Neurosciences, School of Medicine, Cardiff University, UK.
