# Supplementary material for: Adjustment to short-term imprisonment under low prison staffing
Source: BJPsych Bull. 2020 Aug;44(4):139–44. doi: 10.1192/bjb.2020.2 (PMC8058876; doi:10.1192/bjb.2020.2)
Supplement: Supplementary file 1 [file S2056469420000029sup.zip › S2056469420000029sup001.docx]

| **Category** | **Possible additional categories** | **Supporting statements & who refers to it** |
| --- | --- | --- |
| **Routine** | **Components of routine:**  **Education**  **Gym**  **Other exercise**  **Job/work**  **Courses/PACT**  **Social/association**  **Get up**  **Cuppa**  **Fag**  **Food/breakfast/lunch**  **Nap**  **TV**  **Activities**    **Cleaning**  **Games**    **Church**  **Shower**  **Medication**  **Time in cell**  **Visits**  **Phone call**  **Reading**  **Mental Health**  **Writing Letters**  **Physical Health**  **reflection**  **routine**  **Writing Poetry**  **Check emails** | **208** *(education courses look a bit basic- not interested in them),* **209** *(I’m up to date with everything – good education in jail over the years. I couldn’t read or write before prison),* **224** *(I was doing some art classes in the morning and the afternoon),* ***230*** *(maths in the morning…afternoon work – for me that it English),* **231** *(I’ve done health and safety level 1. I’m doing level 2 now. I’ve got an exam on Thursday),* **244** *(haven’t been to education or work or anything like that),* **251** *(There’s courses like music/ education but I don’t need any of that),* **255** *(I want to finish my CV before I go out this time),* **261** *(don’t do much ed. Here)*  **205** *(no gym),* **208** *(hate gym),* **212** *(looking forward…)*, **216** *(every Tuesday),* **217** *(can’t go to the gym),* **219** *(haven’t been to the gym yet)*, **225, 229, 230, 236** *(I’ve been going to the gym since I’ve been in),* **239, 242** *(I’m too lazy for the gym),* **248, 259, 260** *(I haven’t had my gym induction because I keep missing it),* **264, 266**  **205, 206, 208** *(like keeping fit in society e.g. going for long walks)*, **212** *(football),* **219** *(I just lie back on the turf though),* **223** *(half an hour in the yard if that),* **225** *(football),* **230, 242** *(I haven’t done exercise either cos I don’t like football),* **247** *(and just maybe play tennis or something)*  **200, 201** *(I don’t work),* **203** *(I’m being productive at work – I’m hitting all my targets),* **205, 206, 207** *(put in for three jobs),* **208** *(I turn up for work and get turned away- employment issue as with the country as a whole – too many people, not enough work to go around),* **210** *(I’m working in the computer shop- I enjoy it and learnt a couple of thing..),* **211, 213** *(don’t work..),* **214, 215, 216** *(just working- that’s about it really) ,* **218,** **219** *(haven’t done any work yet),* **220, 221, 223, 224** *(I’m waiting for…a cleaning job),* **225** *(they have offered me work but I don’t want it),* **226, 228** (*I’ve put in for work but it hasn’t happened yet. I want to do cleaning on health care),* **229, 230, 232** *(I want to be a cleaner but I haven’t got long left),* **233,** **234, 235** *(I’m supposed to do this afternoon but blagged it. I’ll put an app in to switch),* **240, 243, 244***(haven’t been to education or work or anything like that)*, **245** *(I can just work it back now doing two days a month…)*, **246, 247, 248, 250, 251, 252** *(not working at the moment),* **254** *(just done my work induction),* **256** (*not working),* **257** *(I was working in the laundry but there were too many people in there…),* **258, 259, 260** (*I’m trying to get work),* **261** *(applied for a job),* **262** *(waiting to get a job),* **263, 264, 265,266**, **269**  **200** *(brick laying course),* **203** *(I’ve spoken to pact and I may be seeing my kids at the end of the month…),* **205** (*BICS course and progress to work now),* **219** *(can’t do a computer course but am wanting to do recycling instead),* **222** *(resettlement),* **224** *(I am waiting for a music course),* **225** *(I would rather do courses.. I’ve signed up to a relationship course starting in 2 to 3 weeks – Yeah PACT I’ve signed up with them) ,* **232** (*I’ve done my health and hygiene course and BICS),* **239** *(I’ve done loads of courses before),* **241** (*finished doing the traffic course),* **251** (*There’s courses like music/ education but I don’t need any of that),* **262** *(I want to do the train course, to work on the railways, but everyone wants to get on that),* **263** *(I’m doing a cleaning course too)*  **200, 203, 204, 213, 214, 222, 223,225, 226, 230, 244, 245, 246** (*don’t have association),* **249, 252, 257** *(I don’t use association time),* **264, 267**  **203, 204, 205, 206, 213, 215, 218, 219, 228, 230, 234, 235, 243, 245** *(get up for meds),* **246, 249, 250, 252, 254, 258, 260, 262, 265, 267,**  **205, 218, 219, 222, 230, 234, 235,245, 246, 249, 265**  **205, 228, 245, 265**  **203, 204, 206, 213, 214, 219, 228, 230, 232** (*I’m always eating),* **242, 243, 245, 246, 249, 250, 254, 258, 264**  **200** *(sleeping in the morning),* **204** *(go back to bed),* **207** *(sleeping is the hardest part),* **214, 223** *(sleeping mostly. My head is shot),* **225** *(try and sleep the day away),* **230** *(I’m in bed by 10.30!),* **231** *(I didn’t sleep well yesterday),* **238, 244** *(sleep most of the day),* **257** *(I haven’t been sleeping that well),* **261** *(sleep all morning),* **266** *(I go to bed at about 9.30),* **269** *(sleeping)*  **203, 204, 210, 213, 220, 225** *(there is better TV at night),* **230** *(I’m fed up of TV. There’s nothing to watch),* **231***(watching soaps…I don’t watch tele on the out, I get enough fit in here),* **234** *(soaps to pass the time),* **245** (*I organise my life by the telly now),* **249** *(Have no TV),* **250,** **256** *(I was staying in the cell watching TV really),* **261,264** *(I just watch TV…),* **265, 267, 269**  **209** *(I clean),* **212** (*I just clean really)*, **218** *(cleaning my cell),* **225** *(mop the cell),* **247** *(clean cell),* **250** *(clean cell),* **252** *(clean cell)*  **221** *(play pool),* **231** *(playing pool),* **243** *(play pool),* **245** (*play pool),* **249** *(don’t play pool)*  **230** *(I go to church on the weekend on Saturday morning),* **262** *(I do go to church on Sundays, I light my candles for everyone, my nan and gramp and for my mum cos she’s not well)*  **205, 213, 221, 222, 225, 243,245, 246 , 247, 249, 250, 260, 265,**  **206, 218, 219, 237** *(I haven’t been taking antidepressants),* **245, 265** *(when they open doors rush to meth queue, that’s a priority)*  ***200*** *(chilling in the cell in the afternoons),* **201** *(I’m in my cell for most of the day),* **205** *(cell work),* **217** *(I’ve just been sat in my cell doing nothing) ,* **219** *(I spend all my time sweltering in my cell),* **221** *(just sit in my cell),* **232** *(lying around),* **245** *(get up for meds, then go back to my cell),* **254** *(stay in cell til afternoon),* **256** *(I was just staying in the cell watching TV really),* **257** *(I don’t like to leave my cell…I spend most of my time in my cell- I don’t use association time),* **262** *(I don’t do much in the days really apart from sit in my cell),* **264, 267**  **200** *(visits off my mam and my missus a few times as well)* **209** (*don’t bother with visits – too much for her (mum) – having two hip replacements) ,* **214** *(It’d be good if she could come to visit),* **218** *(I don’t want her [mum] visiting because she isn’t very well),* **220** (*My brother is going to visit and bring my nieces),* **227***(I’ve had no visits),* **247** *(sometimes visit – once a week),* **249** *(I have a visit sometimes-I’ve got one today),* **258** *(I’ve had some visits too, my kids and my family and that; that breaks up the days and weeks),* **259** *(I’ve had some visits in the afternoons which breaks it all up a bit),* **266** *(I’ve had a few visits as well, from my mates and some family members; I get one a week now),* **267** *(I’ve had some visits from my partner too)*  **212** *(I only just put phone credit on now I know I’m going to here for at least another two months),* **215** *(I make phone calls every day),* **225, 240, 243, 249** *(phone my missus),* **252** *(phone missus)*  **217** *(read the prison policies),* **230** *(read the bible),* **249** *(I’ve been reading recently, cos I’ve been on basic and have no TV),* **264**  **222** *(I still haven’t seen mental health),* **248** *(been seeing the psychiatrist every month and the psychologist)*  **212** (*I think I’ve written about two letters- I know some people like doing it but can’t really be bothered),* **213** *(I would write letters to my girlfriend but they won’t let me contact her now, it’s just a bit shit),* **237** *(I’m writing to probation and that),* **240** *(I’ve been getting letters),* **242, 268** *(reading old letters in my cell or new letters or whatever)*  **230** *(I’ve been back and forth to doctors but only diagnosed for the first time),* **232** *(next appointment is Friday)*  **213** (*So I just spend loads of time thinking),* **253** *(learned stuff about myself)*  **207** *(got to get into a routine),* **210** (*even TV is scheduled for you and in the routine)*, **214** *(no same thing every day),* **215** *(you get into a routine and tell yourself it’s not forever)*, **222** *(I’m getting back into it),* **227** (*Exactly the same. Same old routine)* **228** *(same routine every day just over and over),* **251** *(usual daily routine involves fuck all. I don’t do anything),* **253** *(Feel more healthy. I love routine so I like the routine of prison,)* **266** *(same as normal really)*  **268** *(I was writing poetry the other day)*  **215** |
| **Banged up** | **Trapped** | **203, 210** *(locked up),* **213**, **215** *(lock up),* **221, 223** *(23hr bang up down there with half an hour in the yard if that),* **226** (*lock up isn’t until 7),* **228, 230, 231** (*they called for bang up yesterday),* **241, 246, 249, 250, 252, 258, 259** (*I get banged up for lunch),* **261** *(lock up at 4.30),* **264**  **219** *(I spend all my time sweltering in my cell),* **258** (*which means I can be out of the cell for most of the day – thank god – I couldn’t be banged up in there all day; it would do my head in being stuck in the cell as much as some of them are),* **259** *(It’s much better now I’m working – I’m cleaning on the wing so you’re allowed out of your cell for most of the day, it’s much better now)* |
| **Limitations** | **Prison condition as limitations**  **Personal health as a limitation**  **Other personal limitations**  **External limitations** | **203** *(I’ve spoken to pact and I may be seeing my kids at the end of the month. they have to get through social services first. Then if my ex says I can it will be at the end of this month. To be honest that’s the only thing that keeps me going in here),* **207** *(put in for three jobs but haven’t heard anything back…the main thing for me was to get a job but that hasn’t happene*d), **207** *(it’s hell in here-kicking doors- bunch of kids…and the seagulls, keys and kicking doors- 24 hours of noise here!),* **213** (*I would write letters to my girlfriend but they won’t let me contact her now, it’s just a bit shit),* **213** *(Yeah, they are recording my phone calls when I speak to my mother and stop me seeing my girlfriend, that’s pissing me right off),* **214** *(I only work part-time now though. I would like more but I have to wait),* **214** *(I haven’t see my girlfriend yet as there is still the restraining order. It’d be good if she could come to visit.),* **217** *(I’m frustrated because I didn’t get a job. You read the prison policies and they say you must work and I’m begging for it. I’ve spoken to the officers- that blonde officer working today is really helpful, she has been chasing it and said she would send an email. I’ve put in three apps so far…),* **217** *(It’s really hard to get anything done in here. You don’t get any correspondence- it would not have taken very long for the governor to write on the bottom of the slip why I didn’t get a job),* **219** *(I can’t do a computer course but am wanting to do recycling instead),* **219** *(I haven’t been to my gym induction yet cos it’s the same time as canteen so I’ve missed it),* **220** *(I had issues with getting a single cell when I first came in),* **220** *(I only get a hour out when I get back,* **223** *(half an hour in the yard if that),* **223** *(23hr bang up down there),* **228** *(I’ve put in for work but it hasn’t happened yet. I want to do cleaning on health care. Hopefully it will start soon if they sort it out!),* **229** *(When I wasn’t working I got to go to the gym 6 times a week. Now I’m working I can only go 3 times. It’s crazy. It’s £18 a week for nothing though),* **230** *(wait for the door to open at 7.30),* **230** *(I’m on the resettlement wing…most people can’t leave this wing because we owe debt to other people in prison or use drugs…),* **231** *(it’s so loud on the wing),* **237** *(I’ve been putting in requests and heard nothing),* **241** *(doing by head in with all this bag up),* **242** *(the nights are terrible though…),* **243** *(sometimes they let us cleaners out in the evening as well to have a shower),* **245** *(like a drink…I usually brew it in prison…I got caught before I did anything…extra 18 days for it),* **246** *(I don’t have association because I’m in work in the morning and afternoon),* **247** *(You don’t seem to get anywhere when you put the applications in- we made a complaint but I haven’t heard anything about that either. You try to do the right things and put the paperwork in but you just don’t get anywhere),* **251** *(I’ve put into work on recycling and the stores but I haven’t heard anything back),* **257** *(cause you get into trouble if you don’t go),* **260** *(I’m trying to get work but its taking ages. I’ve asked for loads of jobs but no-one I ask knows what they are doing so all I do is bum around),* **260** *(I haven’t had my gym induction because I keep missing it),* **261** *(applied for a job –haven’t heard anything yet- they don’t do much ed. Here- another reason I want to go to [other prison]),* **261** *(I’m just waiting to get a job. I’ve applied for everything but they still won’t give me a job. I want to do the train course, to work on the railways, but everyone wants to get on that. I don’t mind what I do though, I’ve applied for everything, I’ll do anything),* **269** *(There’s nothing to do),* **269** *(I asked to do music but they put me there[talking about laundry])*  **217** *(I can’t go to the gym because I came in with bad ribs),* **217** (*It was really bad when I first got in. I was only on [wing identified] for one night and most people stay for a week or so. I didn’t have an induction because I was detoxing and being sick- the officer said he would come and tell me the information later but I never saw him again… It’s really hard to get anything done in here. You don’t get any correspondence- it would not have taken very long for the governor to write on the bottom of my slip why I didn’t get a job. I just want to know what’s going on)*  **230** *(I go to church on the weekend on Saturday morning so only can go only once),* **234** *(I don’t like going out…the sun pisses me off),* **257** *(I don’t really do much. I don’t like to leave my cell…I don’t like being around crowds of people)*  **226** *(I’ve done nothing over the [bank holiday] weekend- it’s been a long weekend with no work),* **213** *(I don’t work cos they want me to do [can’t read job] and I’m not doing that),* **203** *(…then if my ex-wife says I can it will be the end of this month),* **229** *(Probation have let me down. one was meant to come in and didn’t, another is on maternity, and they have given me a third one. I’ve not met them yet. They weren’t meant to send to send me to a hostel in October and now they are saying December. They are messing me around. It’s doing my head in),* **232** *(I want to be a cleaner but I haven’t got long left),* **237** *(it’s the same as last time. I was out on 25^th^ May and I had nowhere to go. They tried to make me stay in this house with loads of crack heads below. I wasn’t going to stay there. They said there or nowhere. That’s blackmail to me. )* |
| **Boring** |  | **201** *(just the same old, same old),* **208** *(Dull. Boring. I turn up for work and get turned away – employment issue…),* **214** *(same thing every day),* **216** *(it’s been slow),* **217** *(it’s been alright. It’s really boring and frustrating – I’m frustrated because I didn’t get a job…),*  217 (*My brain needs to focus on something),* **223** *(I hate it but I need something to do),* **224** *(Not a lot. Not a lot you can do. Just the same as everyone else),* **225** *(I try and sleep the day away),* **226** *(I’ve done nothing over the [bank holiday] weekend- it’s been a long weekend with no work),* **227** *(I’s just been exactly the same, like the same day every day. It’s the boredom. It’s not hard, it’s boring. Not seeing your family),* **228** *(Boring! Not much else to say to be honest!),* **230** *(fed up of TV),* **251** *(usually daily routine involves fuck all. I don’t do anything),* **258** *(I just get up and work, it been really boring really!),* **260** *(There’s nothing to do),* **268** *(there isn’t much to do)***, 269** (*there’s nothing to do)* |
| **Doing your head in** |  | **207** *(it’s hell in here-kicking doors- bunch of kids…and the seagulls, keys and kicking doors- 24 hours of noise here!),* **217** *(…I said I would kick off in a week if I didn’t get something but my partner said its not worth it. I’ve only got two weeks left),* **229** *(Probation have let me down. one was meant to come in and didn’t, another is on maternity, and they have given me a third one. I’ve not met them yet. They weren’t meant to send to send me to a hostel in October and now they are saying December. They are messing me around. It’s doing my head in),* **241** *(Not too bad. Doing my head in with all this bang up),* **258** *(it would do my head in being stuck in the cell as much as some of them are)* |
| **Busy** |  | **203** *(I’ve been productive at work, I’m hitting all my targets),* **235** *(bit of running around)*, **241** *(makes the week go quicker),* **257** *(I don’t like being around crowds of people. I spend most of my time in my cell – I don’t use any association time. I don’t like being out there with everyone, it’s too busy),* **265** (*I need something to make me think a bit),* **262** *(I’ve applied for everything-I’ll do anything).* |
| **Socialising /Prison Relationships** | **Positive socialising/relationships**  **Negative socialising/ friendship failures** | **200** *(chilling in the cell in the afternoons and then see the other boys when we have social then) ,* **209** *(spend time with the boys),* **217** *(I’ve spoken to the officers- that blonde officer working today is really helpful, she has been chasing it and said she would send an email),*  **217** *(My cell mate got transferred out. It didn’t make sense because he only had a little bit of his sentence left. They moved him further away from where is he from as well. I was a bit sad and I’m not sure why they did it but we will keep in touch when we get out as we are both from the same area),* **222** *(speak to some of my mates),* **223** *(My partner’s cousin is on this wing and can keep an eye out for me),*  **230** *(We all watch the soaps every night. You hear them all when Corrie ends. If you get anyone from jail on Master Mind and give them Corrie as their topic, they would do amazing!),* **231** *(talking to others),* **242** *(talk to some of my mates),* **242** *(my support in here has been mainly from [cell mate]; if it wasn’t for him I’d be lost),* **245** *(Well like a drink, I always have. I’ll be honest with you; I usually brew it in prison when I’m in. Well the other day one of the boys on the wing had brewed in his cell and I had a glass, then I asked him and he gave me a kit so I could do it myself),* **260** *(chat to some boys),* **265** *(then chit chat until I go to work),* **265** *(the boys here are a bit more with it – we talk about current affairs – in the other shops they just moan about the conditions)***, 268** *(spend time standing on the landing chatting to others. Some men come and chat in the cell)*  **207** *(it’s hell in here- kicking doors, bunch of kids),* **213** *(And some of the officers and fucking idiots; rude as fuck and really arrogant. Some are okay, like Mr [name removed] but most of them are wankers),* **219** *(There’s a lot of fighting on [wing identified] at the moment; it wasn’t like that the last time I was here maybe it’s cos there are a lot a kids in now, a lot of them are under 21. That’s why I don’t spend a lot of time out of my cell; it’s violence all of the time),* **231** *(It’s been mental on [wing identified]. They called for bang up yesterday and two guys started fighting. It happened so quick. An officer got punched in the face 3 times. Need to keep an eye out),* **237** *(No one gives a crap in here. There’s no care in the community. I don’t want to be walking the streets again. I want to be transferred out),* **237** *(that’s blackmail to me),* **238** *(staff don’t care. Turn a blind eye to it. I told them I was going to hang myself and told me to do it quietly),* **249** *(I don’t play pool on the landing or anything; bloody all fighting over a pool cue; bloody ridiculous)***257** *(I don’t like being around crowds of people. I spend most of my time in my cell – I don’t use any association time. I don’t like being out there with everyone, it’s too busy)* |
| **Stress** |  | **241** *(So called best mate went around my girlfriend’s house to do odd jobs. He’s a bit of a slag. He slept with 10 women in one week. He was telling her we won’t last when I get out. He is why I am in here for. He started a fight in the street. That’s been stressing me out),* **249** *(I’ve just had a close visit thing; I don’t know why yet, something to do with a closed article [?], I don’t even know what that means; they better tell me soon or I’m going to end up on the block, they will have to ship me out if here)* |
| **Events** |  | **211** *(Nan passed away when I first came in and someone from the Chapel came and another man- I don’t know where he is from... he was wearing all black…I don’t know but he asked if I was alright and if I was going to do anything stupid while I was here but I’m not),*  **218** *(I found out my mum’s ill- she’s got cancer and her organs are failing. I don’t want her visiting because she isn’t very well. I’ve asked for a cancer test as it’s in my family. My granddad died from lung cancer when I first came in),* **220** *(I’m still with my girlfriend so that’s good),* **231** *(Keeping my head down. Talking to others I’ve released that I’ve got a lot more than them. I have a house and daughter and I don’t want to lose that. I need to stop drugs. I don’t want my daughter growing up knowing I’m a smack head),* **234** *(I had court yesterday and it was the first time I went out. Pissed me off. Thought I might be going home. I got 30 weeks, do 15. I’ve already done 6),* **241** *(So called best mate went around my girlfriend’s house to do odd jobs. He’s a bit of a slag. He slept with 10 women in one week. He was telling her we won’t last when I get out. He is why I am in here for. He started a fight in the street. That’s been stressing me out),* **245** *(Well like a drink, I always have. I’ll be honest with you; I usually brew it in prison when I’m in. Well the other day one of the boys on the wing had brewed in his cell and I had a glass, then I asked him and he gave me a kit so I could do it myself. But I got caught before I did anything with it. They gave me an extra 18 days for it but cause I’m on remand I can’t get any extra time so I can just work it back now doing two days a month, then after my sentence then I won’t get any extra time cause I will have worked it all back already. So I won’t have any punishment then when my sentence is finished, as long as I have good behaviour now),* **248** *(I’ve gotten in contact with my brother, last family left. Don’t want to lose him like mum and dad last time. I’m expecting 10 years),* **249** *(I’ve just had a close visit thing; I don’t know why yet, something to do with a closed article [?], I don’t even know what that means; they better tell me soon or I’m going to end up on the block, they will have to ship me out if here),* **258** *(Not really. Apart from my girlfriends’ brother hanging himself),* **262** *(Just my mother being in and out of hospital and not being able to get hold of her. That upsets me then, and then I lose my money omen the phone too and then I can’t speak to anyone else)* |
| **Sense of ability to make choice** |  | **213** *(I don’t work cos they want me to do [can’t read job] and I’m not doing that),* **214** *(I quit recycling- too dirty for me…I would like more but I have to wait),* **218** *(I used to do morning work but mornings aren’t for me. Now I do afternoons),* **219** *(There’s a lot of fighting…that’s why I don’t spend a lot of time out of my cell),* **224** *(I stopped because I don’t like them and I’m waiting for a music course or a cleaning job),* **225** *(I would rather do courses.. I’ve signed up to a relationship course starting in 2 to 3 weeks – Yeah PACT I’ve signed up with them),* **231** *(Keeping my head down. Talking to others I’ve released that I’ve got a lot more than them. I have a house and daughter and I don’t want to lose that. I need to stop drugs. I don’t want my daughter growing up knowing I’m a smack head.),* **232** *(I want to be a cleaner***), 235** *(I’ll put an app in to switch),* **257** *(. I was working in the laundry but there were too many people in there, and I don’t like being around crowds of people. I spend most of my time in my cell – I don’t use any association time),* **259** *(when you’re a cleaner your cell door is unlocked all day, so you can be in and out of your cell),* **262** *(I’m just waiting to get a job. I’ve applied for everything but they still won’t give me a job. I want to do the train course, to work on the railways, but everyone wants to get on that. I don’t mind what I do though, I’ve applied for everything, I’ll do anything)* |
| **Money** |  | **229** *(it’s £18 a week for nothing though)* |
| **Nothing has changed** |  | **237** *(nothing happens. It is the same as last time),* **237** *(I’ve been putting in requests and heard nothing),* **243** *(nothing has happened),* **247** *(You don’t seem to get anywhere when you put the applications in- we made a complaint but I haven’t heard anything about that either. You try to do the right things and put the paperwork in but you just don’t get anywhere),* **251** *(I’ve put into work on recycling and the stores but I haven’t heard anything back),* **260** *(I’m trying to get work but its taking ages. I’ve asked for loads of jobs but no-one I ask knows what they are doing so all I do is bum around),* **266** *(same as normal really)* |
| **Parent** |  | **207** *(just not sleeping here- thinking about the kids),* **231** *(I have a house and a daughter and I don’t want to lose that…I don’t want my daughter growing up knowing I’m a smack head)* |
| **Should be with family** |  | **207** *(just not sleeping here- thinking about the kids),* **227** *(not seeing your family), 236 (The judge was going to do my girlfriend for perverting the course of justice so I had to do without her. I didn’t want her to get crossed examined. Had to protect her but now I’m still here),* **248** *(gotten in contact with my brother, last family left. Don’t want to lose him like mum and dad last time),* **262** *(just my mother being in and out of hospital and not being able to get hold of her. That upsets me then…)***, 268** *(Missing my little boy- if I didn’t have him it wouldn’t be half as bad)* |
| **Chill out** |  | **200** *(chilling in the cell in the afternoons) ,* **232 (***Lying around)* |
| **Drugs** |  | **203** *(then for tobacco),* **220** *(.I had issues with getting a single cell when I first came in and I was coming off the drugs. I used to use cannabis a lot so now I find it hard to relax but my head is clearer. It has its ups and downs. I’ve got a single cell now),* **230** *(Most people can’t leave this wing because owe debt to other people in prison or use drugs like spice and Subutex),* **231** *(I need to stop drugs. I don’t want my daughter growing up knowing I’m a smack head),* **237** *(I never used to touch powder until 2 and a half years ago. It helps you keep walking the streets. I know a guy who will buy me a couple of pints),* **239** *(not thinking about drink or drugs),* **245** *(Well like a drink, I always have. I’ll be honest with you; I usually brew it in prison when I’m in. Well the other day one of the boys on the wing had brewed in his cell and I had a glass, then I asked him and he gave me a kit so I could do it myself. But I got caught before I did anything with it),* **253** *(Helped me get off drugs),* **255** *(I was just drinking all the time I was out),* **256** *(Been on basic for the past few days – cell mate caught with hooch in cells),* **262** *(I can do it this time I think, like stop all the drugs and drinking. My sisters are really pleased with me; they’re starting to get proud of me)* |
| **Felt like Shit** |  | **237** *(fucking shit. I’m getting a bit depressed. I haven’t been taking antidepressants)* |
| **Settled** |  | **201** *(just the same old, same old),* **211** *(Nothing really. It’s alright- I found it easy. I thought it would be worse than this. I work mornings and afternoons),* **214** *(no same thing every day),* **220** *(It’s better now. I’ve adjusted),* **221** *(alright. just getting on),* **222** *(I’m getting back into it),* **242** *(it’s getting better though…not like it was when I first came in),* **240** *(Alright. Not been too bad. Working in multi skills doing carpentry. It’s alright. Good…I was a bit down and that but I’ve got used to it. Well as used to it as you can be),* **248** *(I’m not fighting and stuff anymore; I’ve started to settle, going to the gym),* **263** *(I’m over the cold turkey now)* |
| **Smoking ban** |  | **229** *(It doesn’t bother me. It’s not there. I can’t go down the shop to buy any),* **230** *(There are 2 types of patches and depending on the type you can either peel it off or put it on the kettle to melt it off. Then they use Bible paper. It’s not healthy at all. So many are doing it. I don’t think the government or prison realise how bad it is. It’s crazy. They are for sale. It’s £2 for a patch. Some people are on 50p per day and get into debt. It’s crazy. You can’t smoke prison teabags- only PG tips. They are selling them as well. Prison teabags don’t burn as good. It’s more like powder, not granules. They are lighting it with plug sockets, using razors and a bit of shower gel. If you don’t get it right, the power trips for the whole row of 6 cells. Then no one has power all night. Someone is going to get themselves electrocuted),*  **231** *(Yeah I have but I’m alright now. I’m going to stop when out. It’s hard at first but I wanted to give up. I’ve stopped using patches now. They only give you 10 weeks for free but I don’t use them anymore),* **232***(alright, I used patches),* **233** *(I’m not affected at all by the smoking ban. I gave up a while ago),* **234** *(Fucking horrible. On patches for 4 days. I came off them- they gave me nightmares. I was doing alright but someone showed me how to do the nicotine thing with the patches. I’ve smoked about 4. I was really doing well and it was easier. I’ve got to get back to that.),* **235** *(fine really don’t bother me anymore),* **236** *(At first I was struggling. I’m doing better now),* **239** *(I gave up before the ban),* **240** *(not been too bad),* **241** *(don’t bother me. I’m not smoking when I get out)* |
| **Staff** |  | **213** *(And some of the officers and fucking idiots; rude as fuck and really arrogant . some are okay, like Mr [name] but most of them are wankers),* **217** *(I’ve spoken to the officers- that blonde officer working today is really helpful, she has been chasing it and said she would send an email),* **217** *(the officer said he would come and tell me the information later but I never saw him again),*  **217** *(it would not have taken very long for the governor to write on the bottom of my slip why I didn’t get a job. I just want to know what’s going on),* **229** *(Probation have let me down. one was meant to come in and didn’t, another is on maternity, and they have given me a third one. I’ve not met them yet. They weren’t meant to send to send me to a hostel in October and now they are saying December. They are messing me around. It’s doing my head in),* **230** *(Staff don’t know anything. The [name of team] team or inmates give you better answers),* **231** *(An officer got punched in the face 3 times),* **238** *(staff don’t care. Turn a blind eye to it. I told them I was going to hang myself and told me to do it quietly)* |
| **Out of prison** |  | **215** *(just taking every day. Just behaving- trying to get out),* **217** *(My brain needs to focus on something. When you first get in, you try and block out the outside because you’ve got weeks in here but now I’ve got two weeks left all I’m thinking about is home. My head isn’t great; I’m all over the place. I can’t wait to see everyone but I’m also nervous),* **217** *(we will keep in touch when we get out),*  **226** *(looking forward to getting out),* **231** *(I don’t watch tele on the out),* **231** *(I have a house and daughter and I don’t want to lose that),* **234** *(I don’t like going out. I’m meant to be going to Thailand in September. The sun pisses me off. I had court yesterday and it was the first time I went out…),* **236** *(expected to go home yesterday…its my own fault really. I was so naughty when I was younger. Catches up when I am older),* **236** *(I want to do a boxing match when I get out).* **234** *(Thought I might be going home),* **237** *(I’m trying to get a place when I get out. I’m writing to probation and that),* **237** *(it’s the same as last time. I was out on 25^th^ May and I had nowhere to go. They tried to make me stay in this house with loads of crack heads below. I wasn’t going to stay there. They said there or nowhere. That’s blackmail to me. ),* **237** *(I’m 47 and I don’t want to be walking the streets),* **255** *(It was starting to piss me off being here, I was glad to get out when I did. Before I was out I was doing pre-release stuff with education like doing my CV and that stuff. I want to finish my CV before I go out this time. I was only out for 7 days! I was just drinking all the time I was out. I was recalled for missing my probation appointment…I was supposed to be there at 12.30 but my bus wasn’t until 12…how am I supposed to get all the way to Blackwood in that time? I was pissed when I saw the notice for them looking for me on Facebook so I wrote on it and said “catch me if you can”. Everyone was going on about it then, then I had a phone call saying that it was Sky News and they wanted to interview me for £200. I sent my brother first to check the police weren’t there and he couldn’t see any so I went down…then when the guy form went to shake my hand he snapped my wrist and threw me on the floor…they were the police!),***241** *(he was telling her we won’t last when I get out)* |
| **Violence** |  | **207** *(it’s hell in here-kicking doors- bunch of kids…and the seagulls, keys and kicking doors- 24 hours of noise here!),* **217** *(I said I would kick off in a week if I didn’t get something but my partner said it isn’t worth it),* **219** *(There’s a lot of fighting on [wing identified] at the moment; it wasn’t like that the last time I was here maybe it’s cos there are a lot a kids in now, a lot of them are under 21. That’s why I don’t spend a lot of time out of my cell; it’s violence all of the time),* **231** *(It’s been mental on [wing identified]). They called for bang up yesterday and two guys started fighting. It happened so quick. An officer got punched in the face 3 times. Need to keep an eye out. I didn’t sleep well yesterday. It’s so loud on the wing),* **248** *(I’m not fighting and stuff anymore; I’ve started to settle, going to the gym),* **249** *(I don’t play pool on the landing or anything; bloody all fighting over a pool cue; bloody ridiculous)* |
| **Mental Health** |  | **211** *(he asked if I was alright and if I was going to do anything stupid while I was here but I’m not),* **217** *(My head isn’t great; I’m all over the place. I can’t wait to see everyone but I’m also nervous),* **220** *(I used to use cannabis a lot so now I find it hard to relax but my head is clearer. It has its ups and downs),* **222** *(I still haven’t seen mental health),* **230** *(I got a diagnosis. PTSD. I’ve been back and forth to doctors but only diagnosed for the first time. They said they would be back to see me 4 weeks later but I haven’t seen them since),* **236** *(feel sorry for myself today),* **237** *(I’m getting a bit depressed. I haven’t been taking antidepressants. I don’t want to go down that route. The doctor offered them to me in here but I said no),* **240** *(when I first came in I was a bit down and that but I’ve got used to it. Well as used to it as you can be),* **242** *(It’s been okay through the days; the nights are terrible though. With everything flying through my head. It’s getting better though. It’s not like it was when I first came in),* **248** *(Been seeing the psychiatrist every months and the psychologist),* **251** *(Up and down. Depression and anxiety comes and goes; I just try to sleep the time when I’m feeling like that),* **251** *(I’ve been having bad nightmares; it takes me 2 hours to get to sleep. My anxiety and depression makes them happen I think),* **257** *(I haven’t been sleeping that well. I only get between four and six hours a night and it takes me ages to get off to sleep. I can’t drop off until 12 or one in the morning. It makes me tired),* **263** *(I feel great now, much more energetic and less down, I feel more like myself. I’m over the cold turkey now, I feel great)* |
| **Physical Health** |  | **217** *(I didn’t have an induction because I was detoxing and being sick),***218** *(I’ve asked for a cancer test as it’s in my family),* **223** *(my head is shot),* **222** *(I’ve been ill with a bad stomach and that since I’ve been in. They’ve taken bloods and might send me to hospital for a scan. It worries me- I don’t want to eat but I feel like I have to. I feel anxious because of it),* **230** *(I got a diagnosis. PTSD. I’ve been back and forth to doctors but only diagnosed for the first time. They said they would be back to see me 4 weeks later but I haven’t seen them since…. I think everyone’s health is going to go downhill. They are all smoking patches with teabags),* **232** *(I’m losing weight rapidly. I was 75.5 when I came in. I was weighed down with the doctor at 63.2 because I thought I had lost weight. They are going to look at my food intake but I’m always eating. I’ve spoken to the nurse and she can see I’m losing weight. Next appointment is Friday),*  **237** *(I’ve got arthritis in my feet and hands),* **253** *(feel more healthy),* **255** *(Just what I’ve said about when I was on the out – I’m famous now! My wrist is fucked now from the police when they arrested me…they’ve given me this strap thing but they’ve said that they are going to sort it out for me to get it in plaster in the week now),* **263** *(When I started sleeping better, was waking up fresher so that’s been better. Leg bothering me but nothing I can do about that. I feel great now, much more energetic and less down, I feel more like myself)* |
| **Detox** |  | **217** *(It was really bad when I first got in. I was only on [wing identified] for one night and most people stay for a week or so because I was detoxing and being sick)* |
| **Don’t know what’s going on** |  | **217** *(I just want to know what’s going on),* **217** *(it didn’t make sense),* **217** *(not sure why),* **229** *(they are messing me around),* **230** *(if you don’t push for answers, you never get them),* **247** *(you just don’t get anywhere),* **249** *(Just had a close visit thing, don’t know why yet…they better tell me soon)* |
