# Supplementary material for: Adjustment to short-term imprisonment under low prison staffing
Source: BJPsych Bull. 2020 Aug;44(4):139–44. doi: 10.1192/bjb.2020.2 (PMC8058876; doi:10.1192/bjb.2020.2)
Supplement: Supplementary file 1 [file S2056469420000029sup.zip › S2056469420000029sup002.docx]

| **Category** | **Possible additional categories** | **Supporting statements & who refers to it** |
| --- | --- | --- |
| **Routine** | **Components of routine:**  **Education**  **Gym**  **Other exercise**  **Job/work**  **Gardening**  **Courses/PACT**  **Social/association**  **Prayer**  **Get up**  **Cuppa**  **Fag**  **Food/breakfast/lunch**  **Insulin**  **nap**  **TV**  **Activities**  **(Cleaning)**  **(Games)**  **(Painting cell)**  **(Drawing)**  **Shower**  **Medication**  **Time in cell**  **Visits**  **Phone call**  **Reading**  **Mental Health**  **Writing Letters**  **Church**  **Physical Health**  **Reflection**  **Tobacco**  **Routine**  **Withdrawal** | **100** *(morn and afternoons),* **108** *(library),* **114** *(education a few times),* **124** *(they put me on induction yesterday. Done my maths and English. I did alright, better than high school- they said its equivalent to B GCSE. I’ve done it before when I’ve been in but I did it again anyway),* **127** *(I go to music)*, **133, 138, 140** *(library),* **143** *(put an app in for education),* **154** *(I want to do courses and get a qualification),* **159** *(I want to do a course or something)*  **100** *(Gym Tuesday, Thursday afternoon and Sunday Morning),* **102, 105, 108, 116, 118, 120** *(don’t go to gym)*, **125** *(4-5 times a week),* **126, 127** *(not going to the gym) ,* **128, 131** *(I don’t bother with the gym),* **137, 143, 144,146** *(have to wait to have operation on hand before I can go to the gym),* **147**  **100** *(do some workouts in my cell),* **106** *(clean when they were at exercise),* **107** *(go to the yard to exercise),* **109** (*football on the Astroturf sometimes, not into weights…I’d rather have a kick about),* **113** *(no exercise),***117, 120, 131** *(exercise in my cell),* **137 , 149, 152** *(I don’t bother going out for exercise now),* **157, 160** *( I go in the yard and walk around there sometimes)*  **101** *(better now I’ve got a job),* **102** *(I was working in tea packing but now I’m doing the tools for change course),* **103, 104, 105, 106, 107** *(still waiting for work),* **108 109** *(haven’t been working yet),* **103** *(I do the gardening),* **110** *(find a cell to paint…),* ***111****(I put my name down for tea packing and a course but I haven’t heard anything),* ***112, 113,* 114** *(not going to work),* **116** *(trying to get work*), **117** *(I don’t do work),* **118, 119, 120, 122, 123, 125, 126, 130, 131, 134** *(I do haircutting on the wing),* **134** *(I’m also the smokers champion- I give people advice on coping strategies, just like being a listener really. I’ve not given up yet but plan to next week),* ***135*** *(I had two different jobs),* ***135*** *(I need to go get canteen. I want to work recycling – more fresh air and doing something),* **136, 138** *(I can’t wait to work) ,* **139, 141,** **142** *(before I’d work until 12),* **143** *(I don’t think they want me working),* **144** *(painting cell 29 and painting the block)* **, 145** *(I aint working),* **146** *(I had a cleaners job),* **147** *(I’m waiting for them to get me back into work)* **, 150, 152 , 153, 154** *(I don’t want to just work for the prison),* **155, 158** *(clean cells),* **159** *(working in tea-packing – I don’t like it though)*  **103** *(gardens…nice to get some fresh air and get out and about),* **134** (*I’m a gardener too and work there in the mornings and the afternoon. I check for slugs and feed the plants)*  **102** *(but now I’m doing the tools for change course… I’ve spoken to PACT and I’m going to be doing stuff with them next week),*  **113** *(on tools for change now),* **116** *(resettling course about debt, housing),* **129** *(tools for change),*  **137** (*I was on a PACT course but I came to it and I got kicked off- some security risk or something),* **141** *(I have to do courses before printing),* **147** *(I was on a course but stopped since I moved here because of the split regime. It was road skills),*  **154** *(I want to do courses and get a qualification),* **159** *(I want to do a course or something)*  **100** *(social 11-6 on weekdays),* **101** *(when I get back there is association),* **103, 105, 109** *(association 8am until 11.50),* **111, 114** *(don’t like association with young people),* **115, 117** *(in context of fights),* **120, 121, 122, 128, 129** *(don’t bother with association)*, **136** *(…so they were getting two associations*, **141, 142** *(socialising),* **145, 152, 153, 154** *(I miss association cos it’s in the morning and I’m over here),* **156** (*Assoc. in PM),* **157, 159, 160**  **105**  **103, 104, 110, 115, 116, 121, 126, 128, 130, 133, 141, 149, 150, 151, 153, 156, 157, 158, 159, 160**  **101, 103, 121, 126, 128, 141, 146** *(coffee with the boys),* **157**  **103, 121, 129, 134** *(I’ve not given up yet but plan to next week)*  **104, 105, 113, 115, 126, 128, 129, 131** *(I work on servings as well – I give out the food),* **136, 139, 141, 142, 145** *(fasting),* **146** *(one hot meal a day, don’t have breakfast, tiny portions of cereal. Lunch around 11.30, baguette roll…),* **149,150, 151, 152, 155, 156, 157, 158, 160**  **105**  **105, 127, 128, 139, 141, 143** *(I’ve slept a lot),* **152, 156, 157**  **103, 104** *(I just watch TV in the nights)*, **105, 107, 119, 121, 122, 139, 141, 143, 148** *(he’s down the block now- no TV),* **151, 156, 157, 160**  **117** *(taking part in activities and stuff),* **120** *(I’m doing something different everyday),* **127** *(I go to music),* **141** *(fuck around on wing before work)*  **110, 112, 121, 128, 131** *(Clean! I clean my cell in between cleaning in the morning and afternoon),* **151, 158**  **117** (*playing pool with the boys),* **119** *(maybe a game of snooker or pool),* **128** *(in association I play table tennis),* **142** *(pool),* **143** *(pool),* **152** *(pool),* **155** *(wait for the pool balls to come out…I play table tennis too, but not as good at that)*  **110, 131** *(I’ve also painted my cell…It looks better now. It took all day, I did two coats)*  **143**  **105, 106** *(in context of work),* **113, 121, 128, 129, 136, 141, 145, 150** *(wash my hair),* **151, 152, 153, 155, 157, 159, 160**  **112** *(I’ve started taking Antabuse they did a test and my liver is fucked),* **116** *(wake up, get meds…),* **127** *(I’m waiting to see a doctor on the 14^th^ about my medication and heart),* **133, 148** *(waiting for my meds),* **156, 160**  **114** *(helpful spending time in cell),* **101** *(I more or less stay in the cell – I just call back and forth for coffees),* **107** *(I’m just sat in the cell most of the time),* **111** *(I mostly stay in the cell…),* **111** *(I come out to see some of my buddies….but that’s it),* **112** *(I’ve been keeping myself to myself),* **115** (*like to stay in my cell. Don’t want to be out and about too much),* **124** (*If I’m honest I’ve just been lying in my pit),* **129** *(I only come out of my cell for food and shower),* **142** *(now I’m sacked I stay in my cell),* **149** *(hang around in the cell for a bit),* **151** *(I don’t really leave cell),* **156** *(stay in bed in the AM),* **156** *(but I don’t go out of cell- two boys I speak to on the wing),* **160** *(TV in my cell)*  **117** *(had some visits),* **118** *(visits from partner and mother),* ***120*** *(I’ve had two visits. My ex-partner brought my mum, children and niece. Met my niece for the first time),* **121** *(had a couple of visits),* **125** *(my family brought their colds with them),* **130** (*My brother’s girlfriend and my nephew are planning to visit),* **137** *(visits off my mother),* **139** *(once a week of my mum),* **140** *(had some visitors in),* **158** *(I have visits as well; I’ve had around five and I’ve got one tomorrow: my stepdad’s coming)*  **105, 118, 119, 120** *(use the phone in association time. I’ve put credit on this week for the first time),* **121, 131, 146** (*No option to speak on the phone every day to girlfriend, mum and daughter),* **149** *(phone call them [children] if I’ve got enough phone credit),* **153, 155** *(I would have calls but waiting for my credit),* **156** *(I look forward to my phone call with my girlfriend in the evenings),* **160**  **120** *(I’ve been reading – not done that since I was a kid. Picked a book up and it’s a good story actually),* **150, 156**  **134** *(I’ve seen mental health today- let them know my frustrations. She is going to help me. I don’t think I should be here),* **143** *(I haven’t been doing a lot. I’ve been withdrawing off Valium. I finished last week),* **158** *(saw MH lady and I talked about it)*  **117** *(letters, phone calls, gym…),* **135** *(And I’ve been writing letters to friends and family),* **140** *(writing letters),* **146** *(other cleaners have a letter every day),* **149** *(write letter to my children),* **150, 156** *(I read and write my letters),* **158** *(having letters from my ex. Not writing them anymore)*  ***140*** *(been to the church)*  **147** (*I’m waiting to see the dentist)*, **127** *(I am waiting to see a doctor on 14^th^ about my medication and heart)*  **156** *(I use my time to reflect, think about the past and my mental illness if that’s what you would call it)*  **155** *(get some tobacco if I haven’t got any)*  **137** *(normal routine)*  **143** *(I’ve been withdrawing off Valium)* |
| **Banged up** | ***Trapped*** | **100** *(banged up at 5, from 9 in the morning we are out, though),* **104** *(and then bang up),* **109** *(Banged up most of the time …),* **109** *(but the rest of the time it’s banged up),* **113** *(back for lockdown),* **115, 117** *(locked down),* **121, 138** *(banged up all the time),* **141, 144** *(there’s been a lot of lock up. It’s a pain for them! The officers!)*, **149, 154, 160**  **106** *(I needed to get out of the wing),* **107** *(I’m just sat in the cell most of the time …. It would be different if there wasn’t a telly, but it’s OK),* **108** *(It’s OK – better than being stuck in the cell),* **108** *(People I’m not getting on with really),* **115** *(let out for 10 minutes),* **130** *(They only moved me here yesterday. I want to move off here. I can’t sleep and it’s loud on here. I’m anxious- I’m anxious anyway but it is making me more so. I keep scratching),* ***141*** *(wait for them to open the door),* **148** *(they’ll take you to the block for anything…no TV, nothing, probably no blanket),* **149** *(wait to be unlocked),* **159** *(I only do it to get me out of the cell. I want to change to something else)* |
| **Limitations** | **Prison Conditions as Limitations**  **Personal Health as a Limitation**  **Other Personal Limitations**  **External Limitations** | **100** *(I do some workouts in my cell, but there is only so much you can do),* **105** (*Sunday’s gym sometimes when we get a chance),* **107** *(I’m still waiting for work. put in three job applications but they haven’t been back to me),* **111** *(I put my name down for tea packing and a course but I haven’t heard anything),* **112** *(The person who is teaching us is on holiday),* **116** *(use gym as much as I can. Not a lot really),* **117** *(I don’t do work now cos I filled in the forms and I’m waiting for them to give me something),* **117** *(when the buzzers go off and theres a fight on the wing, that pisses me off cos we are locked down. Yesterday there were 15 of us shoved in a cell. It pisses me off. It happens around 3 times in every association),* **120** *(they wouldn’t let me go to dad’s funeral on [date given]. I was a bit upset – Dad was a bit of a twat anyway. Don’t always see eye to eye with my Dad’s side of the family),* **122** *(I’ll get a job when I get sentenced- can’t be fucked like),* **123** *(trying to get a job but they are stopping me. I said I was racist and now they are stopping me. I’ve put in a form so they can take it out- I only said I was racist so I didn’t have to share a cell with no drug head… They were going to send me to shop 4 but there’s been no work for 3 weeks),* **130** *(They only moved me here yesterday. I want to move off here. I can’t sleep and it’s loud on here. I’m anxious- I’m anxious anyway but it is making me more so. I keep scratching),* **135** (*I need to go to get canteen. I want to work in recycling- more fresh air and doing something),* **136** *(Depending on who is on we get a shower when we are back),* **136** (*There is a bit of tension because some officers were letting all the kitchen workers out so they were getting two associations. People were saying it’s not fair),* **137** (*I was on a PACT course but I came to it and I got kicked off- some security risk or something),* **138** *(I can’t wait to work. I’ve been asking everyday),* **139** (*Not required today- I’m not sure why, think they are short staffed),* ***141*** *(I’m wasting time on the computer really. You have to do courses on there before printing but I won’t make it past the computer bit),* **141** *(wait for them to open the door),* **142** *(not I’m sacked I stay in the cell),* **143** *(don’t think they want me working. Put an app in for education),* **146** (*No option to speak on the phone every day to girlfriend, mum and daughter),* **147** (*I was on a course but stopped since I moved here and because of the spilt regime. It was road skills. I’m waiting for them to get me back into work),* **147** *(I’m waiting to see the dentist. Remember I had toothache last time you came? Well I’ve got an abscess. I asked to see the dentist but I’ve not heard back. I asked the nurse twice),* **148** *(Healthcare staff…think I’m trying to get drugs. They only give paracetamol. I was pissing blood and passing kidney stones on Monday. There is no help in here. I’ve been in and out for 22 ½ years and it makes your worse. Tick you want to see housing yes or no and no one ever comes),* **150** *(I do my work that they allocate me as quickly as possible so I can do some of my own design stuff then to keep my mind working when I’m in here. Although I think they will start clamping down on that soon),* **150** *(I’m just waiting now to see if I will be going out on tag),* **150** *(being here is the opposite to rehabilitation for me…I’m going to be in a worse position when I get out than when I came in),*  **156** *(I think the system is designed to break you- completely mismanaged as well and poorly run. Why do you get treated better if you’ve foreign – [Ethnic Group]- get 4 meals a day and we get one; how is that fair?),* **158** *(I’m starving; that’s all: the portions are tiny. I’ve been upset at times, I did something stupid to hurt myself),* **160** *(now I’m on basic I can’t)*  **101** *(Won’t let me go to the gym because of my blood pressure),* **127** *(I’m not going to the gym as I’m waiting to see a doctor on the 14^th^ about my medication and heart),* **143** *(I’ve slept a lot. I haven’t been doing a lot. I’ve been withdrawing off Valium. I finished last week. Don’t think they want me working…might have a fit because of withdrawing. It’s happened before),* **146** (*I have to wait to have operation on hand before I can go to the gym)*  **149** *(phone call if I’ve got enough phone credit),* **155** *(I would have calls but waiting for my credit)*  **101** *(Don’t go out because of the weather),* **130** *(There is a chance I could get out in June. That’s pretty good. I’m waiting for court)* |
| **Boring** | **Same thing/that’s it** | **100** *(Just the same stuff all the time it gets boring*), **104** *(same everyday… it’s just the same every day),* **110** *(Get up, clean, find a cell to paint and that’s it for the day. It stops the boredom but that’s about it),* **112** *(same as ever- monotonous),* **114** *(been better- trying to kill the days)*, **122** *(Fuck all like),* **123** *(Boring)*, **135** *(I’ve been working on computers but I’ve had enough of that…it’s not sometime I’ll be doing when I’m out),* **135** *(I need to get to canteen. I want to work in recycling- more fresh air and doing something)*, **150** (*It’s been pretty dull to be honest, nothing happens)*  **100, 104, 105, 106** *(Nothing has really changed since we spoke last time),* **107** *(I’m still waiting for work, put in three job applications but they haven’t been back to me),* **110, 111** *(I put my name down for tea packing and a course but I haven’t heard anything),***112, 114, 130, 151, 152** *(it’s just been the same old)* |
| **Doing your head in** |  | **100** *(...the same stuff, day in and day out, it just does your head in )* |
| **Busy** |  | **101** *(better now I’ve got a job ….full time now and time is flying),* **106** *(I’ve got a new job now …… it’s busy and I like to keep busy),* **112** *(I work with BICS cleaning, which keeps me busy in the day),* **117** *(times going quicker now I’m doing stuff),* **126** (*I get to work weekends which is good- they always used to go slowly for me. Time goes faster now),* **128** *(look busy),* **131** *(I need to keep busy),* **134** *(Like to keep busy),* **135** *(I’ve been up to all sorts),* **150** *( I do my work as quickly as possible so I can do some of my own design stuff then to keep my mind working when I’m in here),* **158** *(keep myself occupied)* |
| **New opportunities** |  | **101** (better now I have a job), **106** (I’ve got a new job now …… it’s busy and I like to keep busy. Only been here two days now mind), **102** *(now I’m doing the tools for change course),* **102** *(spoken to PACT and I’m going to be doing stuff with them next week),* **106** *(new job now at reception),* **109** *(I’ve just got a job doing washing),* **111** *(I put my name down for tea packing and a course),* **113** *(tools for change),* **116** *(went for induction yesterday- trying to get work now so hoping to hear about that),* **124** *(done my maths and English. I did alright, better than high school),* **134** *(I’m also the smokers champion- I give people advice on coping strategies, just like being a listener really. I’ve not given up yet but plan to next week),* **138** *(I can’t wait to work, I’ve been asking everyday),* **146** *(get to meet new people),***152** *(I’ve started a new job),* **155** *(wait for the pool balls to come out; I’m good at that, I play for a team on the out),* **159** *(I want to change to something else; I want to do a course of something),* **153** *(I’ve been cleared to be a prisoner listener. History of self-harm so surprised, didn’t ever think I would. Looking forward to starting that).* |
| **Socialising /Prison Relationships** | **Positive socialising/relationships**  **Negative socialising/ friendship failures** | **103** *(chill out with the boys and have a chat, the boys are all good* *in here …),* **103** *(I chill out in the evenings with my pad mate - we get on well – we watch TV and we have out little [can’t read]),* **103** *(The guy… who was always shouting has gone down the block, he was really loud so it’s better now),* ***108*** *(work’s a different atmosphere. I’ve gotten to know people but don’t care what they’ve done),* **111** *(I come out to see some of my buddies at association but that’s it),* ***117*** *(I am speaking to other prisoners),*  **117** *(playing pool with the boys and talking to them. I know a lot of them from school and a lot of them are my brothers friends too),* **121** *(talk to cleaners),* **122** *(see the boys on association),* **124** (*I’ve got a friend on here that I grew up with. He is younger than me. He’s in here for selling drugs and normally I wouldn’t hang around with dealers but I’ve know what he’s been through. He’s got 5 years and I feel bad for him. I check in on him and we have a chat. He is younger than me and I feel bad for him you know),* **126** *(My brother is now on the same wing. Been here [wing identified] for 2-3 weeks. I was asking for ages but they took about a month to move him. I’ve asked if he can work in the kitchen with me),* **135** *(I’m sharing with my mate on [wing identified]). I was in with my other mate but he went to [name] prison. I’m gutted. I won’t be seeing him for 3 years – that’s how long he has got left. I’ll have to do another sentence to see him),* **141** *(chat some more),* **142** (*I prefer it to [names prison]. There’s a good group of boys. There is 9 of us from [location]),* **143** *(socialising),* **146** *(get to meet new people…chat to them make you feel more comfortable),* **146** *(drank a lot of coffee with the boys),* **151** *(If I need to ask the guards something I will go out but I don’t wander around the wing or anything),***152** *(I just stay in with the boys and play pool and stuff like that),* **154** (My padmate just got released but we had a good laugh, hopefully a boy I know on the out is coming in with me on Monday), **155** *(wait for the pool balls to come out; I’m good at that, I play for a team on the out),* **155***(I like to check on my padmate because he self-harms so I look out for him- I’ve stopped him twice already. Single cell today but they’ve put him back now),* **156** *(two boys I speak to on the wing),* **159** *(talk to someone on association)*  **100** *(me and my cell mate just end up bugging the shit out of each other),* **101** *(there’s association, but I more-or-less stay in the cell),* **108** (*people I’m not getting on with really [by implication on the wing as work is cited as relieving that],* **103** *(The guy from X [country cited] who was always shouting has gone down the block… so it’s better now),* **123**( *I only said I was racist so I didn’t have to share a cell with no drug head),* **136** *(There is a bit of tension because some officers were letting all the kitchen workers out so they were getting two associations. People were saying it’s not fair),* **148** *(Healthcare staff are fucking bad. They don’t fucking care. They think I am trying to get drugs. They only give paracetamol. I was pissing blood and passed kidney stones on Monday. There is no help here…The young staff got attitude. I heard them saying they’d like to rough up one of the prisoners…),* ***156*** *(people always asking you for something- so I steer clear. Plus don’t like that you have to treat someone like these prisoners (murderers and rapists) with respect – If I do anything then I will be the bad guy so I just stay away)* |
| **Stress** |  | **102** *(I’m stressing a lot, thinking I’m a parent, shouldn’t be here, I should be out there looking after ….),* **117** *(my psoriasis came out because I was stressed),* **130** (*They only moved me here yesterday. I want to move off here. I can’t sleep and it’s loud on here. I’m anxious- I’m anxious anyway but it is making me more so. I keep scratching),* **150** *(My only stress is possibly running out of canteen but that actually never happens)* |
| **Events** |  | **104** *(Nan’s death),* **108** *(ex-partner stole all belongings from home and changed the locks),* **111** *(worried they will send me to [other prison]. There’s a boy there who said he would kill me if he sees me again so I’m worried they will send me there. I have told them here but it’s not the same at [other prison mentioned]. You will always see people at [other prison mentioned]),* **126** *(My brother is now on the same wing. Been here [wing identified] for 2-3 weeks. I was asking for ages but they took about a month to move him. I’ve asked if he can work in the kitchen with me),* **126** *(This sentence is going to be a long one. I lost access to my daughter because of it. I need to change),* **130** (*No… there is a chance I could get out in June. That’s pretty good. I’m waiting for court. My brother’s girlfriend and my nephew are planning to visit. I’m in touch with her. My brother is dead. He hung himself),* **135** *(I’ve had two warnings, been in basic and come off it),* **135** *(I’m sharing with my mate on [wing mentioned] I was in with my other mate but he went to [other] prison. I’m gutted. I won’t be seeing him for 3 years – that’s how long he has got left. I’ll have to do another sentence to see him),* **136** (*That trial I told you about, well he got sentenced to 14 years. It’s not as much as we expected- we were told between 14 and 19 years. I feel like that’s done now and he will be having a hard time. He is still saying he is innocent but more people are coming forward. It sounds like a cliché but now that that’s done, I feel like when I get out it will be a fresh start),* **146** *(trying to break cycle of gangs on the out),* **146** *(The only thing I find is that when I’m in prison and you are innocent and no pleas have been said, the staff shouldn’t judge. They all say that I have to walk away from them. They can’t believe the change in me this time. 90% I’ll have a not guilty- what convictions I have I don’t know what I can do),* **150** *(I need to look after my business [clothing design]. My dad and brother can only cover for me so much cause there’s stuff they can’t do/ Like I have German contacts and they like to be spoken to in German. Not that I can speak German properly but they like to be greeted in German. I’ve got money in the bank at the moment but that will only last so long, it will run out at some point. Being in here is the opposite to rehabilitation for me…I’m going to be in a worse position when I get out than when I came in),* **158** *(having letters from my ex)* |
| **Dreading the outside** |  | **104** (*I don’t want to get out: I’m dreading it. My ex is being funny …….I breached to get me put back in here),* **119** *(concerned about being released- used to being in cell and used to the surroundings then suddenly will be let out. That is the thing I am most worried about),***138** (*normally I’m really worried about the out)* |
| **Sense of ability to make choice** |  | **101** *(they wanted me to do clothes but I am tea packing),* **101** *(I more or less stay in the cell – I just call back and forth for coffees),* **109** *(so I’d rather have a kick about),* **111** *(I mostly stay in the cell…),* **111** *(I come out to see some of my buddies….but that’s it),* **112** *(I’ve been keeping myself to myself),* **114** *(not going to work…helpful spending time in cell),* **115** *(like to stay in my cell. Don’t want to be out and about too much),* **123** *(I am hoping to get a cleaning job),* **126** *(my brother is now on the same wing…I was asking for ages but they took about a month to move him),* **129** *(I only come out of my cell for food and shower),***135** *(I need to go get canteen. I want to work recycling- more fresh air and doing something),* **135** *(I’ve been working in computers but I’ve had enough of that),* **138** (*I can’t wait to work. I’ve been asking every day. I’m hoping to go back to the kitchen. I went for one day before going to the hospital wing. Hoping they will let me go back),* **150** *(I do my work that they allocate me as quickly as possible so I can do some of my own design stuff then to keep my mind working when I’m in here),*  **151** *(If I need to ask the guards something I will go out but I don’t wander around the wing or anything),* **154** *(I want to do courses and get a qualification. I don’t want to just work for prison),* **156** *(stay in bed AM…nothing good on TV then I’ll read and drink tea…I don’t go out of cell- two boys I speak to on the wing. I’ve got everything I need),* **159** *(I want to change to something else; I want to do a course of something)* |
| **More money** |  | **106** *(I get more money over there),* **130** *(They put my money up already to £19 a week. I’m loaded),* **150** *(I’ve got money in the bank at the moment but that will only last for so long)* |
| **Shouldn’t be here** |  | **102** *(shouldn’t be in here),* **134** *(I don’t think I should be here)* |
| **Parent** |  | **102** *(I’m a parent)* |
| **Should be looking after family** |  | **102** *(should be out there looking after my missus and kids),* **149** *(I’m really worried about my children; my son has taken over the house now. He’s 24 but he’s a young 24. He’s got a support worker helping him now and I feel really guilty about that. I know he’s a man at 24 but like I said he’s a young 24 and he’s still my kid)* |
| **Drugs** |  | **114** *(don’t want to be doing any drugs in here),* **148** *(they think I’m trying to get drugs…there was one guy who was hallucinating. He had taken spice. He was thinking the officers were out to get him…he had a panic attack…spice and sub is everywhere. I don’t touch spice),* **150** *(I know who’s who now and I could get drugs and drink if I wanted to but haven’t done it. I feel like I’ve just lost the desire. I’m not interested in the drugs on offer…I’m not going to sniff Subutex off a toilet seat like the rest of them, and I know I couldn’t get enough drink to satisfy me so that’s pointless),* **155** *(get some tobacco if I haven’t got any)* |
| **Settled** |  | **117** *(I’m feeling much better),* **123** *(Settled in more. Before I found it hard, now I think I just get on with it),* **125** *(I’m just getting on with it. I’ve settled in now),* **128** *(I’m settled now. Been in 10 times and got my routine now I’ve been sentenced. As soon as I’m sentenced I’m alright,- I know how long I’ve got and what I’ve got to do),* **138** *(I feel much better. Feel happy),* **138** (*No, it’s brilliant. I feel better and put a bit of weigh on. Best thing that’s ever happened to me. I don’t know if I’ll stay off them when I’m out but I hope so),* **157** *(I’ve got everyone that I need),* **158** *(better now. Started to get up and do stuff, keep myself occupied)* |
| **Smoking ban** |  | **138** (*No, it’s brilliant. I feel better and put a bit of weigh on. Best thing that’s ever happened to me. I don’t know if I’ll stay off them when I’m out but I hope so* ), **139** *(doesn’t bother me. We’ve got patches. I haven’t put them on for two weeks now. Just drilled it into myself that I can’t have a fag so tough luck),* **140** *(I’ve forgotten about smoking now. I’m a non-smoker. Don’t know if I’ll smoke when I get out),* **141** (*Cool. The odd burn here and there. Eating a lot more lollies! If I’m not chewing on my necklace, I’m eating lollies. I’m eating more sweets and crap. Burn used to be the highlight of canteen. Now loads of munch. I spent £20 on munch. I bought a few ecigs but they are crap. I would rather spend the £5 on munch. It was kicking off on the wings on the first week of the ban. Burn nowhere but it’s about now. It’s calmed down to be fair. Everyone has got used to it. You could feel it before, it was proper tense),* **142** *(It’s alright. It don’t bother me. It’s easy),* **143** *(Not good at all. Everyone is smoking tea bags, Bible paper and a bit of glue. Its gives you a bad chest. It’s no good for you. I’ve been thinking about writing a letter to the Gov. to ask for ecigs with the liquid. You can get a proper drag from them. The ecigs we have are rubbish. They don’t work and they run out of charge. I get why they are doing it. If you are a non-smoker, you don’t want to share a cell with a smoker. But if I was a non-smoker, I wouldn’t want to share a cell with someone smoking teabags! The screws can’t manage it),*  **144** *(Fine with it now. I’m past it. Lungs feel clearer and I feel better. I’ve got nothing on my hands. They used to be yellow where I was smoking all the time. When I get out I won’t. Maybe a cheeky one when I have a drink. I know people like that, they only want to smoke when they are out having a drink).***145** *(still smoke electric cig. It helps you cut down),* **147** *(It’s alright),* **148** *(Stressful. Got no choice. All smoking tea bags and plastic. It’s fucking worse for their health. I don’t smoke it)* |
| **Staff** |  | **134** *(I’ve seen mental health today let them know my frustrations. She is going to help me),* **136** *(there was a bit of tension because some officers were letting all the kitchen workers out so they were getting two associations. People were saying it’s not fair),* **144** *(there’s been a lot of lock up. It’s a pain for them! The officers!),* **148** *(Healthcare staff are fucking bad. They don’t fucking care. They think I’m trying to get drugs. They only give paracetamol. I was pissing blood and passed kidney stones on Monday. There is no help in here… The young staff got attitude. I heard them saying they’d like to rough up one of the prisoners. I heard them when I was waiting for meds. They’ll take you to the block for anything),* **151** *(if I need to ask the guards something I will go out),* **158** *(ACCT officers helped me),* **146** *(The only thing I find is that when I’m in prison and you are innocent and no pleas have been said, the staff shouldn’t judge. They all say that I have to walk away from them. They can’t believe the change in me this time. 90% I’ll have a not guilty- what convictions I have I don’t know what I can do)* |
| **Out of Prison** |  | **150** *(I need to look after my business (clothing design),* **134** *(I hate weekends even on the outside),* **104** *(I don’t want to get out),* **135** *(it’s not something I will be doing when I am out),* **136** *(when I get out it will be a fresh start),* **138** *(normally I’m really worried about the out),* **155** *(I play for a team on the out),* **146** *(trying to break the cycle of gangs on the out),* **154** *(hopefully a boy I know on the out is coming in with me on Monday),* **150** *(I’m going to be in a worse position when I get out than when I came in).* |
| **Mental Health** |  | **103** (*I want to move off here. I can’t sleep and it’s loud on here. I’m anxious- I’m anxious anyway but it is making me more so. I keep scratching),* **117** *(Until I spoke to you I’ve felt like shit),* **140** *(My mood has been up and down- worried, depressed, angry),* **148** (*he had a panic attack),* **149** *(I’ve come off my ACCT. I’m feeling a little better now. I’m still anxious but I’m better than I was),* **153** *(History of self-harm),* **155** (*I like to check on my padmate because he self-harms so I look out for him- I’ve stopped him twice already. Single cell today but they’ve put him back now),* **156** *(I use my time to reflect, think about the past and my mental illness if that’s what you would call it).* **158** *(I’ve been upset at times, I did something stupid to hurt myself. Saw MH lady and I talked about it, felt better, ACCT officers helped me.)* |
| **Physical Health** |  | **112** *(my liver is fucked),* **117** *(My psoriasis came out because I was stressed),* **135** *(I feel a bit ill today),* **143** *(I might have a fit because of withdrawing),* **147** (*Remember I had toothache last time you came? Well I’ve got an abscess. I asked to see the dentist but I’ve not heard back. I asked the nurse twice),* **148** *(I was pissing blood and passing kidney stones on Monday),*  **149** *(I’ve been coughing up blood recently, quite a bit of it actually. I’ve been recently anxious about that. I’ve given a sample and I’m waiting for the results now),* **159** *(keep waking up in the middle of the night. Sometimes I have good sleeps but not most of the time. I don’t feel tired in the days…sometimes I get up and feel dizzy then I goes back to normal. The doctor just told me to drink more water)* |
| **I need to change** |  | **126** *(I lost access to my daughter because of it. I need to change)* |
| **Violence** |  | **117** *(when the buzzers go off and there’s a fight on the wing…3 times in every association…),* **141** *(kicking off in the wings on the first week of the ban, burn nowhere but it’s about now, its calmed down. you could feel it before, it was proper tense)* |
